# Aberration correction for low voltage optimized transmission electron microscopy

**DOI:** 10.1016/j.mex.2018.08.009

**Published:** 2018-08-25

**Authors:** Jaromír Bačovský

**Affiliations:** a DELONG INSTRUMENTS a.s., Palackého třída 3019/153 b, 612 00 Brno, Czech Republic; b Institute of Physical Engineering, Faculty of Mechanical Engineering, Brno university of Technology, Technická 2896/2, 616 69 Brno, Czech Republic

**Keywords:** Aberration corrected low voltage transmission electron micrscopy, Low voltage transmission electron microscopy, Aberration correction, Hexapole corrector

## Abstract

Further development of low voltage electron microscopy leads to an aberration correction of the device in order to improve its spatial resolution. The integration of a corrector to a desktop transmission electron microscope with exclusively low-voltage design seems to be a challenging task. The benefits and potential of the Rose hexapole corrector implemented to such a system are critically considered in this paper. The feasibility of miniaturized corrector suitable for desktop LVEM is especially discussed, including the aspect of corrector contribution to chromatic aberration that appears to be crucial. Optimal corrector parameters and resolution limits of such a system are proposed.

• Improved spatial resolution
• Spherical aberration correction
• Permanent magnet transfer lenses

## Graphical abstract

**Figure:**
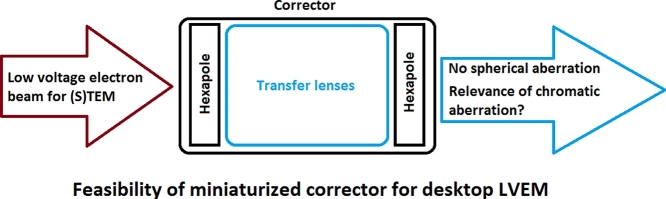

## Introduction

In contrast to advanced light-optical microscopy techniques capable of resolution beyond the diffraction limit, which is considered the physical limit for classical microscopy, electron microscopy has not yet approached this physical limitation. Uncorrected conventional electron microscopy limited mainly by chromatic and spherical aberration is not able to achieve spatial resolution better than 50λ [1].

There are two combinable approaches to resolve smaller objects: reduction of the De Broglie wavelength and correction of aberrations. Although λ itself is small enough even for low accelerating voltage, its reduction has a significant influence on system aberration. On the contrary, increasing accelerating voltage has high requirements for technical parameters of electronics, as well as dimensions of main microscope column and sample stability against radiation damage. The appropriate way to exploit the physical potential of the instruments is to correct the most serious aberrations.

Basic principles of aberration correction have been known for 70 years [2], but practical implementation has been complicated by technological limitations for a very long time. The first generation of correctors called “proof of principle” confirmed the possibility to use rotational asymmetric multipole electronoptical components for aberration correction. However their own imperfections severely deteriorated image resolution. The rapid development of accuracy of mechanical manufacturing and stability of the electronics over the past decades has enabled the practical use of correctors in the most advanced electron microscopes [3]. The current commercially available corrected transmission microscopes work with accelerating voltage in the range of 100–200 kV, but corrected systems with lower energy are still under development.

Low voltage transmission electron microscopy (LVEM) uses an electron beam with an energy of 5–30 kV. The reduction of radiation damage of the sample and contrast enhancement necessary for investigation of sensitive samples containing light atoms and weak bonds can be considered as the main advantage of such systems. Certain technical aspects also allow the LVEM to be constructed in a desktop design, in contrast to the common dimensions of conventional TEM/STEM devices. In this article the feasibility and potential of aberration corrections of desktop LVEM will be discussed with special attention to 5 kV LVEM5 [4] and 25 kV LVEM25 [5] optimized systems developed by Delong Instruments a.s.

There are experimental projects focused on the development and construction of a suitable corrector designed for LVEM [6], [7], [8]. Namely *The Sub-Ångstrom Low-Voltage Electron Microscopy* (SALVE) project from the research group from Ulm University and the Japanese project of Delta corrector should be mentioned. Different approaches to the construction of a corrector were chosen by these groups, though both of them did use readjustment of conventional high voltage microscope for low energy. These solutions for LVEMs are not able to completely profit from the low energy of electrons. Our goal is to investigate of the possible implementation of a compact corrector to the existing Delong LVEM desktop microscope with an exclusively low-voltage design.

## Correctors

Image resolution is mainly influenced by a spherical and chromatic aberration of the objective lens. Correction of these unwanted phenomenons is the first step to improve imaging ability. The effects of other terms in the aberration polynomial are less important. The degree of importance of each aberration is a relevant issue determining the appropriate method of correction.

According to Scherzer's proposals, successful corrections of unavoidable primary spherical and chromatic aberrations of round lenses are based on multipole elements. Various configurations of multipoles with different correction abilities have been proposed [2].

The most successful basic concepts are hexapole and quadrupole–octupole correctors for spherical aberration and a quadrupole–octupole complex corrector for spherical and chromatic aberrations. All concepts have advantages and disadvantages. Their relevancies as a function of electron-optical system parameters are crucial for applicability of the particular correction system.

### The hexapole corrector of spherical aberration

The simplest design of a hexapole corrector consists of two hexapole elements separated by a round-lens doublet. The system of the two hexapoles is inconvenient not only because of its increasing correction power, but it also reduces the intrinsic hexapole astigmatism – a predominant hexapole aberration [9], [10]. A detailed scheme with required positioning of each component can be found in [11].

For the purpose of coma elimination and minimization of the secondary 5th order spherical aberration, enhanced by the combination of a hexapole with the objective lens, there has to be another round lens doublet situated in front of the corrector [12]. This kind of corrector is not able to solve the problem of chromatic aberration. On the contrary, the transfer lenses increase the total chromatic aberration of the electron-optical system. The effect is remarkable especially with the minimization of the corrector for a low-voltage system. The consequences and reasons for this problem to occur will be discussed later.

### The quadrupole–octupole corrector

The quadrupole–octupole corrector of spherical and chromatic aberrations consists of 4 quadrupoles and 2 octupoles in minimal configuration. However arrangements with a different complexity have been published. The most advanced design, called *ultracorrector*, was published by Rose [13]. This complex corrector is assembled by combining two identical multipole multiplets, each consisting of seven quadrupoles and seven octopoles [14]. The universal ultracorrector should be able to compensate for the aberrations up to the third rank inclusively. The possibility of correction of chromatic aberration is an undisputed advantage of such systems, but the complexity of the multipole arrangement leads to some problems regarding mechanical alignment. Due to the higher number of multipole elements, the size of the complete device is significantly larger than the hexapole version. It would be negligible, though, when considering conventional transmission microscope with massive main column. It can, however, be important for smaller desktop LVEMs. The comparable size of the corrector and the main column can lead to some additional mechanical vibrations, which further deteriorate the final resolution. That is all due to the increase in size of the microscope column.

## Parameters of current LVEM 5 & LVEM 25

This paper pays special attention to low voltage microscopes produced by Delong Instruments co. The calculations were made using the parameters of these devices. For clarity, the important parameters as specified by the manufacturer are listed below (Table 1, Table 2)

**Table 1:** Relevant theoretically calculated parametres of LVEM5 and LVEM25. [4][5].

Mode	Accelerating voltage [kV]	$C_{s}$ [mm]	$C_{c}$ [mm]
*LVEM 5*			
TEM	5	0.64	0.89
STEM	5	0.64	0.89

*LVEM 25*			
TEM	25	1.03	1.05
STEM	15	0.80	0.85
STEM	10	0.64	0.72

**Table 2:** Declared experimental resolution limit of uncorrected LVEM 5 and LVEM 25 [4], [5].

LVEM 5	LVEM 5	LVEM 25	LVEM 25	LVEM 25
5 keV	5 keV	10 keV	15 keV	25 keV
TEM	STEM	STEM	STEM	TEM

2 [nm]	2.5 [nm]	1 [nm]	1.3 [nm]	1[nm]

## Estimated resolution of corrected LVEM

The total resolution is determined by the combination of all aberrations, but it is necessary to use only the most important ones to arrive at a relevant estimate. In the case of an uncorrected system the following have to be considered: the primary spherical aberration, the chromatic aberration and the diffraction limit. The contributions of the partial aberration discs $d_{s}$, $d_{c}$ and $d_{d}$ can be summarized to a total aberration disc d:

(.1) $$d=\sqrt{d_{s}^{2}+d_{c}^{2}+d_{d}^{2}}.$$

It can be specified in more detail:

(.2) $$d=\sqrt{{\left(k_{1}C_{s}\alpha_{i}^{3}\right)}^{2}+{\left(k_{2}\frac{\Delta E}{E_{0}}C_{c}\alpha_{i}\right)}^{2}+{\left(0.61\frac{\lambda }{\alpha_{i}}\right)}^{2}},$$

where standard notation is used [15].

The experimental spatial resolutions of the current LVEM 5 and LVEM 25 are given in Table 2. The declared values are in good agreement with theoretical models using the standard evaluation method. From Eq. (.1) it is obvious that the optimal solution of a corrected system is not significantly limited by one major contribution, but all primary contributions should be comparable. The degree of importance of each aberration has been studied in various system conditions to determine which aberration is limiting and thus should be corrected.

The most manifesting aberrations of common electron optical devices are the chromatic and the geometric (especially the primary spherical aberration) ones, their influences on the size of the paraxial space (determined by aperture angle) are competing with the influence of the diffraction limit. An optimal aperture angle therefore exists. The correct choice of the aperture angle is necessary to maintain the best possible resolution.

**Fig. 1:**
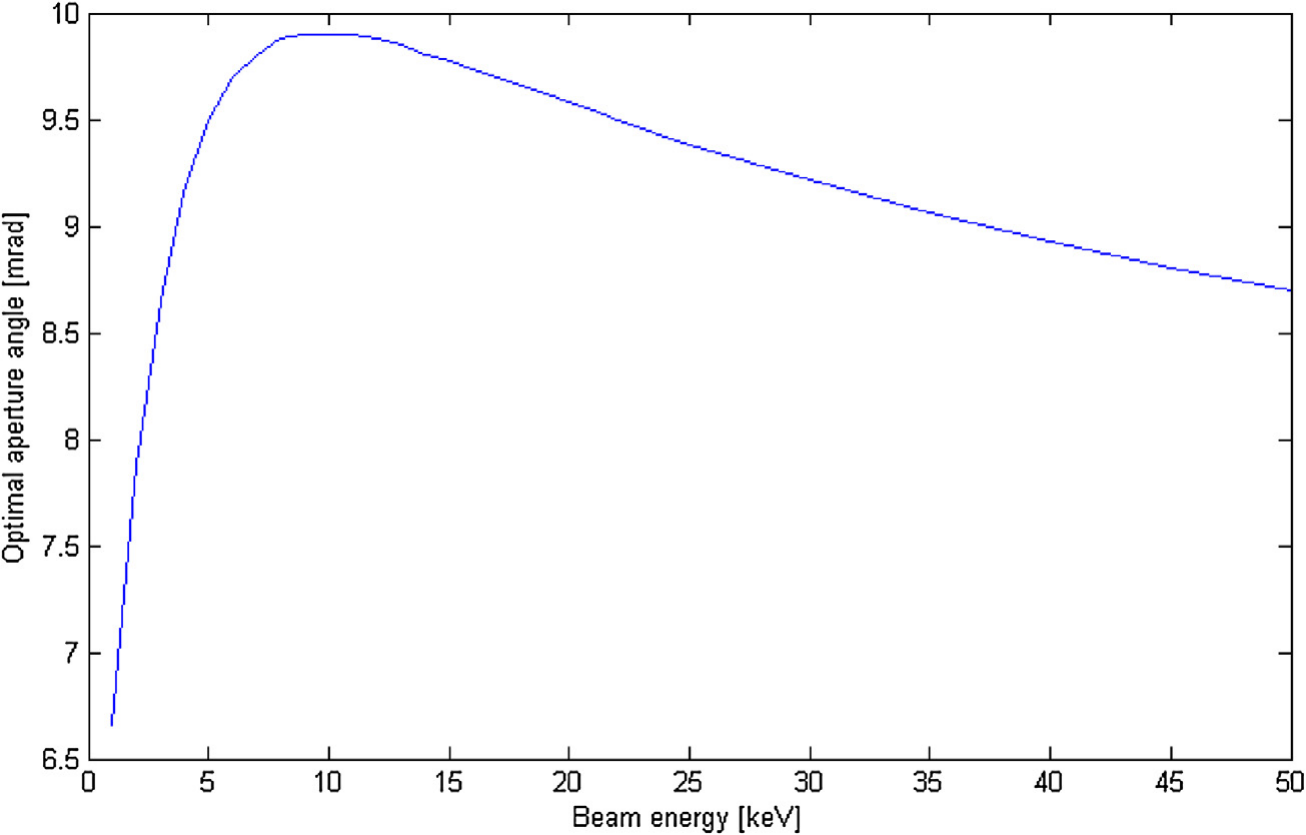
Optimal accelerating energy for a specific set of aberration coefficients (LVEM 5) $C_{s}=0.64$ mm, $C_{c}=0.89$ mm, ΔE=0.6 eV.

Optimal aperture angles $\alpha_{\mathrm{optim}}$ have been calculated in the range of LVEM accelerating voltage. From Fig. 1 it can be seen that for specific a set of aberration coefficients there is preferred accelerating energy. This conclusion can also be obtained directly from the aberration integrals, where the integrands are dependent on the accelerating voltage.

The results for all above mentioned modes of interest with a typical energy spread ΔE of Schottky cathode and specific aberration coefficients (Table 2) are presented in Table 3. The change of the optimal aperture angle for typical energy spreads of Schottky and CFE cathodes is 0.2–1.5 mrad.

**Table 3:** Optimal setting of aperture angles obtained by minimisation of the integral aberration disc.

ΔE [eV]	LVEM 5	LVEM 25	LVEM 25	LVEM 25
	5 keV	10 keV	15 keV	25 keV
0.6 (Schottky)	9.5 [mrad]	10.2 [mrad]	9.4 [mrad]	8.3 [mrad]
0.3 (CFE)	11.0 [mrad]	10.6 [mrad]	9.6 [mrad]	8.5 [mrad]

**Fig. 2:**
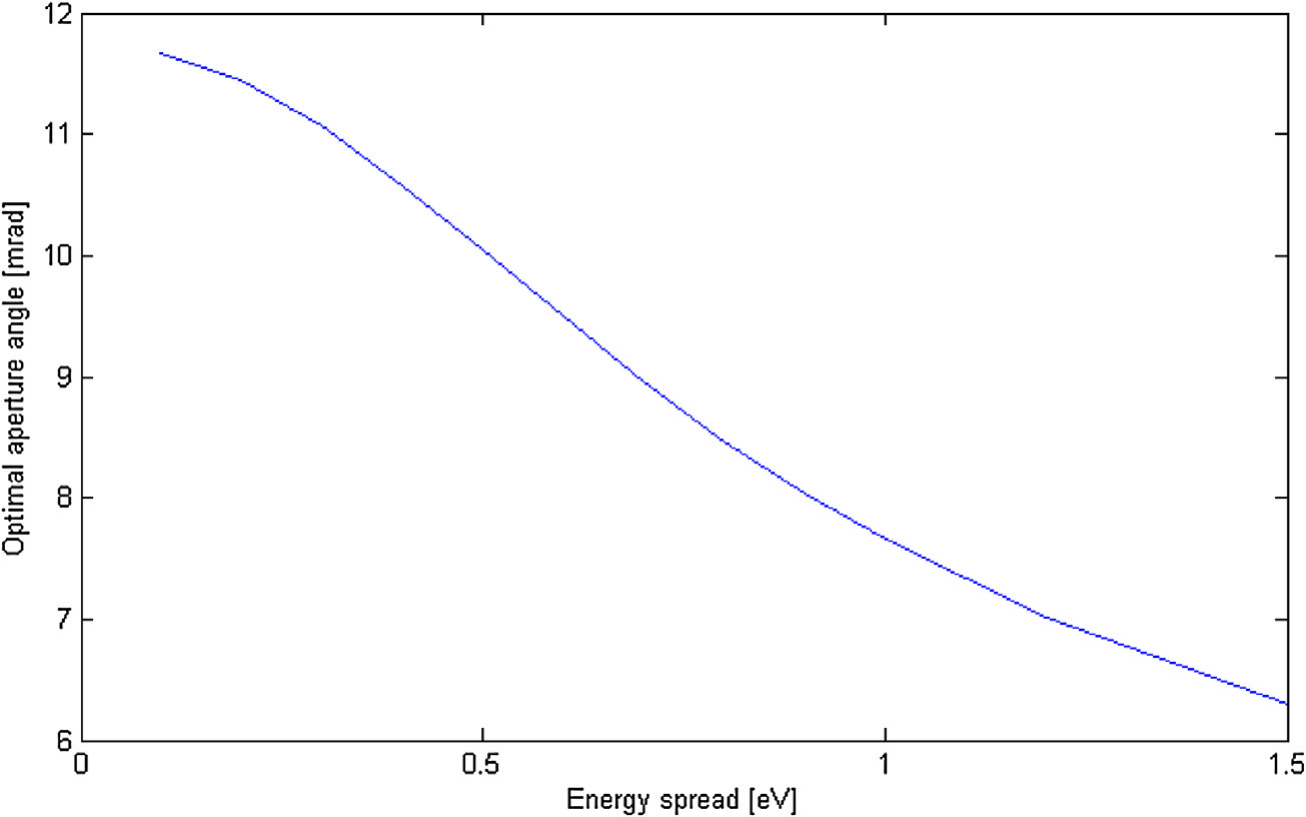
Model parameters $C_{s}=0.64$ mm, $C_{c}=0.89$ mm, E=5 keV.

The change between Schottky and CFE is significantly bigger considering the lower electron energy, all because of higher importance of the chromatic aberration. The effect of the monochromatisation degree is continuously demonstrated in Fig. 2. It can be clearly observed that the lower energy spread leads to a larger $\alpha_{\mathrm{optim}}$ and thus to a larger paraxial space.

The integral aberration discs were calculated with $\alpha_{\mathrm{optim}}$ according to Eq. (.2). It can be considered to be the resolution limit of an uncorrected system. The calculations were done for a Gaussian imaging plane (i.e. $k_{1}=1$, $k_{2}=1$), where the screen is placed approximately (Table 4).

**Table 4:** Integral aberration discs for $\alpha_{\mathrm{optim}}$ in the Gaussian imaging plane ΔE=0.6 eV.

	LVEM 5	LVEM 25	LVEM 25	LVEM 25
	5 keV	10 keV	15 keV	25 keV
d [nm]	1.5	0.92	0.79	0.67

A closer look at the partial aberration discs is helpful in order to understand the behavior of the resolution limit. The data prove that the decrease in the accelerating voltage makes the chromatic aberration more severe. The diameter of the integral aberration disc for the 5 kV LVEM is reduced by CFE to app. 80% when using Schottky cathode. In the case of 25 kV with other identical parameters (LVEM5), the integral aberration disc became smaller by only 4% (5).

**Table 5:** The aberration discs for energy spread typical for Schottky and CFE cathodes (0.6 eV, 0.3 eV). Aberration coefficients were used from LVEM5 and LVEM25 [4], [5].

	5 keV		25 keV	
$\Delta E_{0}$	0.6 eV	0.3 eV	0.6 eV	0.3 eV
$d_{s}$ [nm]	0.27	0.43	0.30	0.31
$d_{c}$ [nm]	1.01	0.59	0.21	0.10
$d_{d}$ [nm]	1.11	0.95	0.56	0.55
d [nm]	1.52	1.20	0.67	0.64

## Wave aberration theory

Geometrical aberrations can also be described by a phase shift χ between an ideal nonaberrated system and a model system with a considered set of aberrations:

(.3) $$\chi (\alpha )=\frac{2\pi }{\lambda }\left(-\frac{1}{2}\Delta f\alpha^{2}+\frac{1}{4}C_{3}\alpha^{4}+\frac{1}{6}C_{5}\alpha^{6}+\frac{1}{8}C_{7}\alpha^{8}+\ldots \right).$$

A different notation is used in Eq. (.3). The subscript i of spherical aberration coefficient $C_{i}$ denotes an order of aberration. A phase shift is also dependent on aperture angle α and the wavelength of used particles λ.

For the purpose of this paper the polynom will be reduced, because of the correction ability of the hexapole corrector, which is able to achieve active correction of the 3rd order and the partial compensation of the 5th order. Furthermore quadrupole–octupole corrector is able to correction up to 5th order spherical aberration (system limited by the $C_{7}$). Thus only spherical aberration up to the 7th order is considered.

To achieve the best possible resolution, it is necessary to find a compromise between the geometrical aberrations and the diffraction limit. To maintain the required image quality, the phase shift has to be below a certain value. The Rayleigh criterion is used as a commonly accepted phase shift. χ=π/2 i.e. P-V (peak to valley) is equal λ/4. A higher phase shift causes low intensity of the 0th order maximum of the diffraction pattern. It results in an increase of the intensity of the higher order maximums and de facto to a loss of the image contrast.

A quantitative evaluation of the image quality was done by Strehl ratio:

(.4) $$S=e^{-{\left(2\pi \mathrm{RMS}\right)}^{2}},$$

where RMS Root-Mean-Square, a parametre which describes the wavefront, is defined:

(.5) $$\mathrm{RMS}=\frac{P-V}{4.5}.$$

Although the definition of the spatial resolution itself is a tricky task and different approaches are described in literature and used by different manufacturers, the generally accepted value of Strehl ratio to maintain reasonable image quality is S≥0.8 [16], [17]. For the purpose of this study the value is set to S=0.88.

**Fig. 3:**
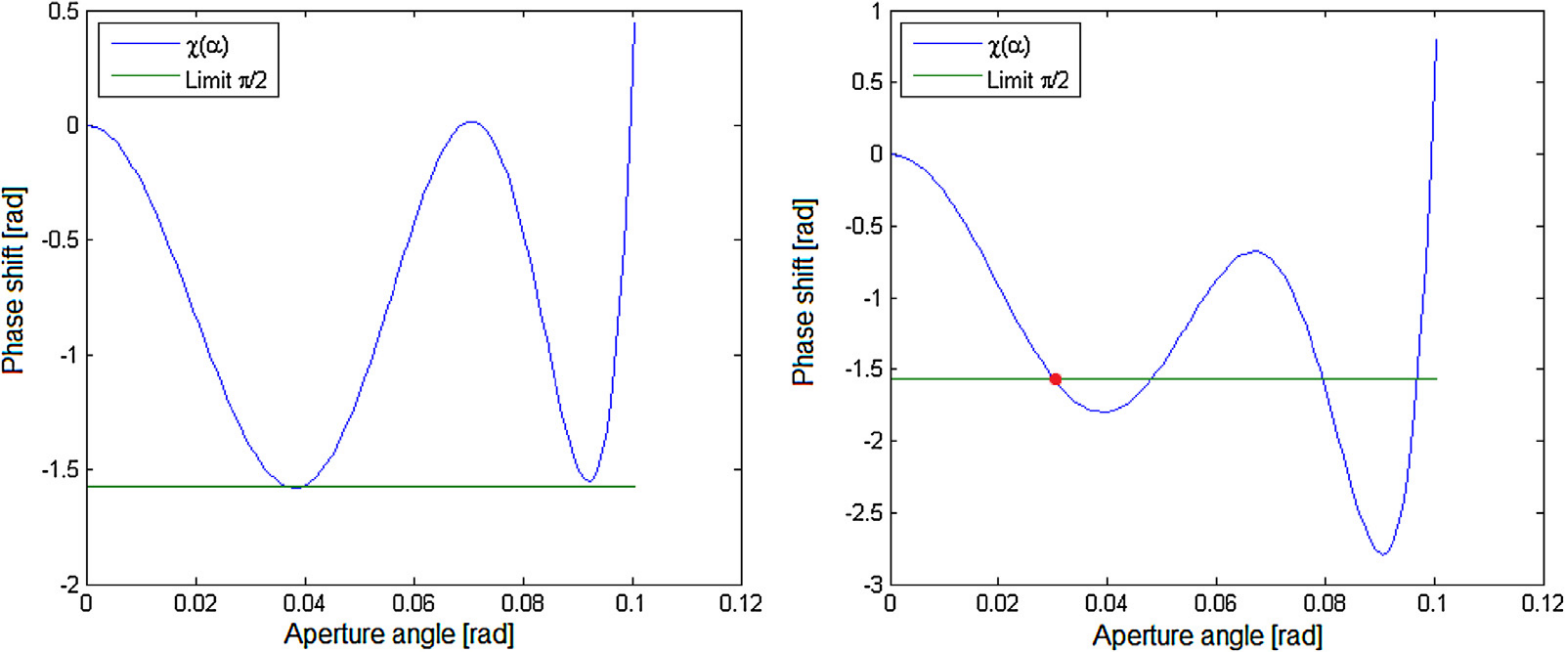
*left*: The phase shift behaviour with an appropriate choice of a coefficient (corrected system). χ(α) stays below a certain limit in the biggest possible interval of aperture angle. *right*: In case of improperly selected coefficients or uncorrected system, the phase shift will exceed (red dot) the limit already with lower α. (For interpretation of the references to color in text/this figure legend, the reader is referred to the web version of the article.)

The goal of eliminating the undesirable effect of aberrations is to achieve a given limit of a phase shift at the greatest possible aperture angle. The behaviour of the polynomial function (.3) determines the existence of local extremes. The required form of the polynomial, which means oscillating phase shift below a certain predefined limit until the last local extreme appears, can be created by the proper choice of coefficients (Fig. 3).

Formulas for the most appropriate coefficients $C_{i}$, defocus, the corresponding resolution and the aperture angle in different conditions were derived analytically (Table 6) by Intaraprasonk et al. [18] and Chang et al. [19]. It should be emphasized that it is not optimal to set coefficients to zero, because the aberrations of a lower order are capable of reducing the influence of the higher order aberrations, which then cannot be principally corrected by the chosen type of corrector.

**Table 6:** Formulas for an optimal setting of the aberration coefficients by the corrector and parameters of systems corrected for the primary (limited by the 5th order) spherical aberration resp. corrected for the secondary (limited by the 7th order) spherical aberration. Term 1/b means limiting fraction of wavelength λ with tolerable effect on the image quality [18].

Order of correction	3	5
Optimal $C_{3}$	$-4{\left(\frac{3}{2b}\lambda C_{5}^{2}\right)}^{1/3}$	$10{\left(\frac{2\lambda C_{7}}{b}\right)}^{1/2}$
Optimal $C_{5}$	–	$-6{\left(\frac{2\lambda C_{7}^{3}}{b}\right)}^{1/4}$
Δf	$-3{\left(\frac{9}{4b^{2}}\lambda^{2}C_{5}\right)}^{1/3}$	$4{\left(\frac{8\lambda^{3}C_{7}}{b^{3}}\right)}^{1/4}$
$\alpha_{\max}$	${\left(\frac{96\lambda }{bC_{5}}\right)}^{1/6}$	$2{\left(\frac{2\lambda }{{\mathrm{bC}}_{7}}\right)}^{1/8}$
Resolution	$0.61{\left(\frac{b}{96}C_{5}\lambda^{5}\right)}^{1/6}$	$\frac{0.61}{2}{\left(\frac{b}{2}C_{7}\lambda^{7}\right)}^{1/8}$

**Fig. 4:**
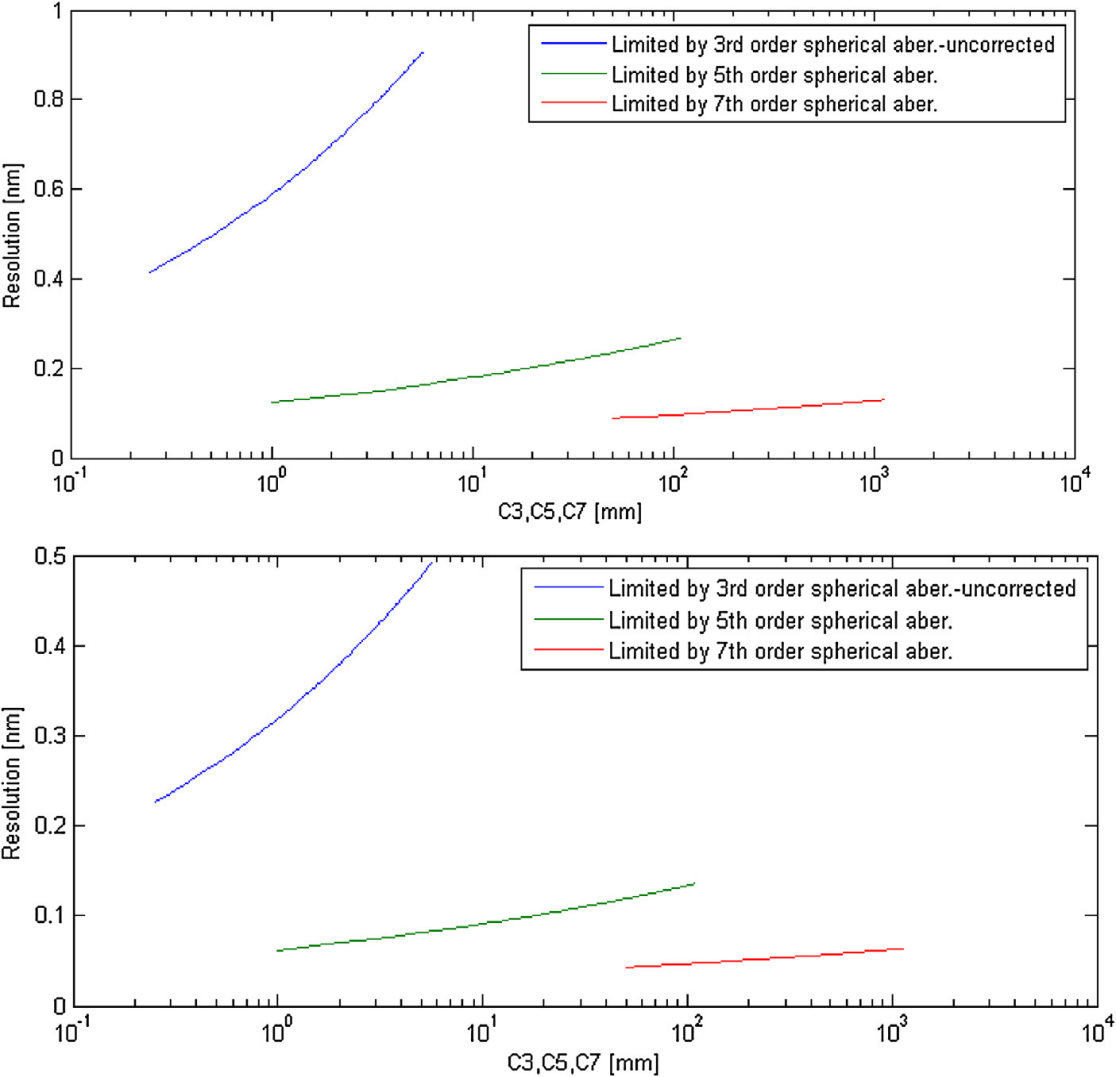
The image shows the dependence of an expected resolution limit on the aberration coefficient of a limiting order of the spherical aberration for a different order of an aberration correction. Non-corrected system limited by the primary spherical aberration (green), correction of the 3rd order spherical aberration i.e. limited by the 5th order of spherical aberration (green) and correction of the 5th order spherical aberration i.e. limited by the 7th order of spherical aberration (red). $E_{0}=5$ kV (upper fig.), $E_{0}=25$ kV (lower fig.). (For interpretation of the references to color in text/this figure legend, the reader is referred to the web version of the article.)

According to the optimal setting of coefficients, the significance of different order correction of the spherical aberration is shown in Fig. 4. The new calculated resolution limits, depending on the value of the coefficient of limiting aberration, can be clearly seen.

The significant effect of active correction of the primary spherical aberration can be observed. The influence of the additional correction of the secondary spherical aberration (the 5th order) theoretically improves the attainable resolution, but requirements for stability of the corrector's power supplies can be too high. In addition, it is necessary to take into account the influence of the chromatic aberration, which will be discussed later.

The discussed model is able to find the optimal setting of the aberration coefficients by a corrector to reach the biggest aperture angle, meeting the chosen requirements of the phase shift. The proper choice of coefficients enables to extension of the aperture angle to the interval after the last local phase shift minimum, as is shown in Fig. 3.

Optimal aperture angles of all LVEM5 and LVEM25 imaging modes were calculated for a system limited by the 3rd and the 5th order spherical aberration (Table 7). The aperture angles would clearly enlarge due to the correction. The aperture angle of an uncorrected microscope is 0.01 rad [4], [5]. A correction of the primary spherical aberration promises to improve this value by at least three times and with the correction of the 5th order spherical aberration, it would be possible to use an aperture angle approximately eight times higher as compared with the current systems.

**Table 7:** Optimal aperture angles of the corrected system: correction up to the 3rd order $\alpha_{3}$ ($C_{5}=100$ mm), correction up to the 5th order $\alpha_{5}$ ($C_{7}=1000$ mm) S=0.88.

	5 keV	10 keV	15 keV	25 keV
$\alpha_{3}$ [rad]	0.040	0.038	0.037	0.035
$\alpha_{5}$ [rad]	0.083	0.079	0.077	0.075

The resolution of the corrected system was calculated with the aperture angles from the table above (Table 7). The results are shown in Table 8. Because of significant enlargement of the optimal aperture angle, the chromatic aberration disc is also bigger. To maintain the diameter of the chromatic aberration disc comparable with the spherical one and therefore take full advantage of the correction potential of the spherical aberration, it is necessary to increase the degree of monochromatisation. Using a new aperture angle and keeping the energy spread without any reduction leads to deterioration of the chromatic aberration disc to $d_{c5}=8.8$ nm in case of LVEM5 with correction of the 5th order spherical aberration. It has been proved that, in order to achieve some meaningful values of $d_{c}$ comparable with $d_{s}$, it is necessary to reduce the energy spread to ΔE=0.08 eV for LVEM 25 and ΔE=0.04 eV for LVEM5 (correction of the 3rd order spherical aberration). The energy spread requirements for the 5th order correction are even higher. In this case, it should not exceed the value ΔE=0.02 eV.

**Table 8:** The resolution of the system with a correction of the 3rd order $d_{3}$ ($C_{5}=100$ mm) and the 5th order spherical aberration $d_{5}$ ($C_{7}=1000$ mm) neglecting the influence of the chromatic aberration. S=0.88.

	5 keV	10 keV	15 keV	25 keV
$d_{3}$ [nm]	0.26	0.20	0.17	0.13
$d_{5}$ [nm]	0.13	0.09	0.08	0.06

The available technology of monochromators is currently capable of fulfilling these conditions in a very limited way, because the energy spread below ΔE=0.1 eV is very difficult to achieve [20]. The chromatic aberration thus still poses a very serious problem.

The estimated resolution of the $C_{s}$ ($C_{3}$) corrected systems with a monochromator and a CFE gun is shown in Table 9.

**Table 9:** Computed resolution for systems with reduced energy spread and accordingly optimized aperture angles. The typical energy spread of ΔE=0.1 eV is used in case of monochromated system ($d_{\mathrm{monochrom}}$) and ΔE=0.3 for cold-field emission gun $d_{\mathrm{CFE}}$.

	5 keV	10 keV	15 keV	25 keV
$d_{\mathrm{CFE}}$ [nm]	1.06	0.56	0.45	0.34
$d_{\mathrm{monochrom}}$ [nm]	0.61	0.32	0.26	0.20

## Chromatic aberration of systems corrected by a hexapole corrector

The previous part of this article dealt with the problem of a spherical aberration correction. There is, however, also a chromatic aberration that can be significantly severe for LVEM, as is shown in Section “Estimated resolution of corrected LVEM”. Unfortunately as was mentioned in the introduction, chromatic aberration is correctable only by a more intricate quadrupole–octupole corrector. A hexapole corrector is not able to correct or even improve the manifestation of this aberration. On the contrary, rotationally symmetric elements, which are part and parcel of a hexapole corrector, add their own contributions to the chromatic aberration of the whole electron-optical system. The minimum configuration of the sextupole corrector consists of a telescopic round lens doublet (transfer lens) and two sextupoles placed in front of and behind the doublet, according to the Rose arrangement [11].

In order to create an aplanat, it is necessary to transfer the coma-free nodal point $N_{1}$ to the coma-free point of the objective. For this purpose another doublet of the transfer lens has to be used.

From the chromatic aberration point of view, only the transfer lenses have to be investigated. The contribution of a separate transfer round lens will be derived then.

The chromatic aberration is usually described by a coefficient $C_{c}$ defined, in case of a magnetic rotationally symmetric lens, by integral [21], [22]:

(.6) $$C_{c}=\frac{\eta^{2}}{4U}\int_{z_{o}}^{z_{i}}\;B{\left(z\right)}^{2}h^{2}\mathrm{dz}.$$

(.7) $$\eta =\sqrt{\frac{e}{2m_{0}}}$$

In case of a thin lens approximation, the integral can be modified to the form:

(.8) $$C_{c}=\frac{\eta^{2}}{4U}\int_{-\infty }^{\infty }\;B{\left(z\right)}^{2}h^{2}\mathrm{dz}.$$

The integration boundaries (−∞;∞) can be used, because the out-of-field parts have zero contribution to the total $C_{c}$. Due to the same approximation concentrating the field into a tiny space, the change of axial ray distance from the axis h inside the lens can also be neglected and replaced by a constant equal to the object distance $z_{o}$:

(.9) $$h(0)=z_{o}.$$

**Fig. 5:**
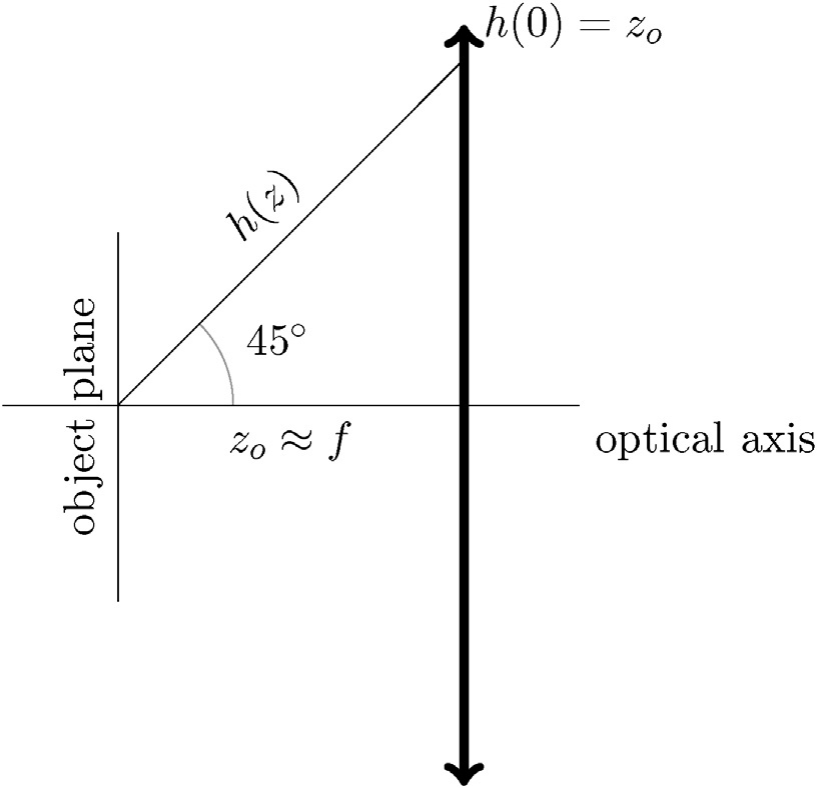
Scheme of the axial ray trajectory through a thin lens placed in the origin of coordinate system. The object is placed on a focal plane ($z_{o}\approx f$) of an arbitrary lens (“arrow notation”). This scheme corresponds to the situation of the usage of the lens as an objective.

This equality relation is determined from definition of an axial ray specified by its position in the object plane $h(z_{o})=0$ and the unit slope in the same plane. The situation is more clearly seen when observing Fig. 5.

Because the distance of the axial ray from the axis is constant in a non-zero magnetic field space of the thin lens, it can be moved in front of the integral:

(.10) $$C_{c}=\frac{\eta^{2}}{4U}z_{o}^{2}\int_{-\infty }^{\infty }\;B_{z}^{2}\mathrm{dz}.$$

The integral is replaced with consideration to the equation for focal length of the magnetic lens f:

(.11) $$\frac{1}{f}=\frac{e^{2}}{8m_{0}E_{0}}\int B_{z}^{2}\mathrm{dz}.$$

Putting Eqs. (.11) and (.10) together:

(.12) $$C_{c}=\frac{\eta^{2}}{4U}\frac{8m_{0}U}{e}z_{o}^{2}\frac{1}{f}$$

simplifying the algebraic expression, the final dependence is obtained:

(.13) $$C_{c}=\frac{z_{o}^{2}}{f},$$

This dependence seems to be in a direct contradiction with the generally accepted rule for the design of an electron optical system. According to well-known guidelines for electron-optical lens design, the chromatic aberration coefficient is approximately equal to the focal length of the lens:

(.14) $$C_{c}\approx f,$$

This rule applies especially to magnetic lenses $\left(C_{c}[\mathrm{mm}]=f[\mathrm{mm}]\right)$. In the case of the electrostatic lenses, there is still a direct variation between $C_{c}$ and f but the coefficient is several times higher due to the difficulties in achieving high gradients. This apparent paradox can be explained by considering the imaging conditions. Usually the chromatic aberration of an objective lens is critical for the resolution of the entire microscope. In this common configuration, the object is located very near the object focus of the lens to reach high magnification and indeed the Eq. (.13) can be transformed to the form (.14), because the object is close to the focus and therefore the distance of the axial ray from the axis in the lens region is equal to the focal length of the lens. In case of the different object positioning along the optical axis, the above-mentioned transformation of Eq. (.13) is not possible and the dependence is clearly inversely proportional.

**Fig. 6:**
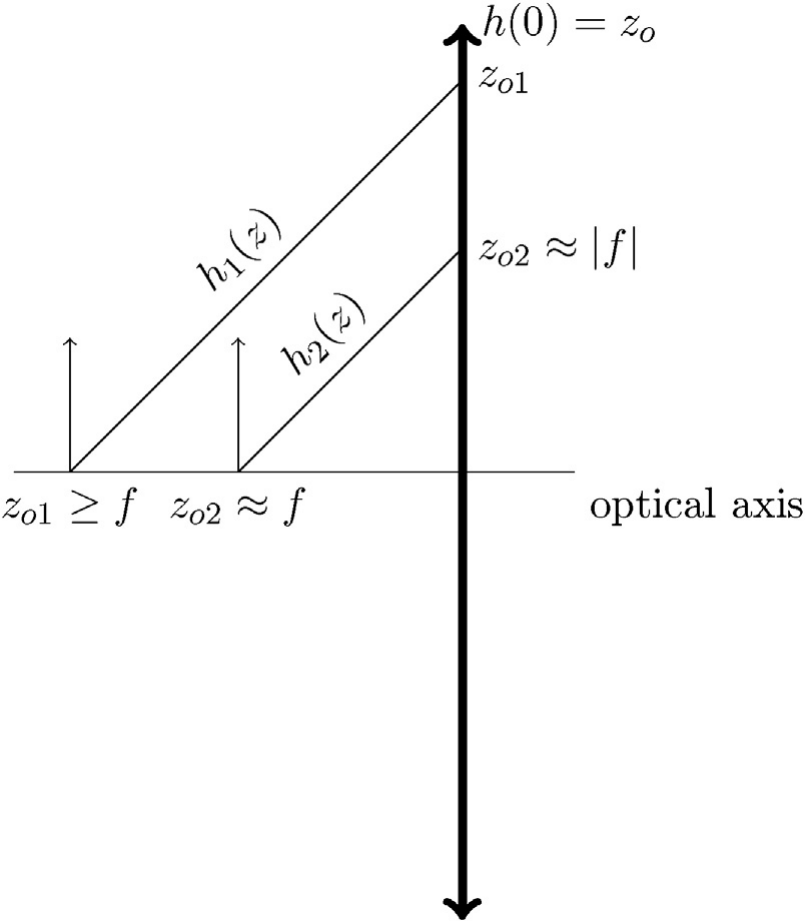
The situation denoted by mark 1 represents a general position of the object out of the lens focus (object distance $z_{o1}$). This situation corresponds to the corrector doublet usage. Label 2 denotes the discussed situation of using the lens as an objective (object distance $z_{o2}$) leading to a direct proportionality between $C_{c}$ and f(.14).

The comparison of both situations is given in Fig. 6, which can be helpful for correct understanding of the behaviour.

The conclusions resulting from Eq. (.13) are very important to the corrector concept. According to the above-mentioned guidelines for the optimal lens design (.14), a lens with the short focal length could be mistakenly preferred. In case of the presence of a transfer lens in the corrector set-up, the contribution of a single transfer lens to the $C_{c}$ can be derived based on Eq. (.13). The distance from the axis of the above-mentioned ray in the transfer lens $h_{\mathrm{TL}}$ is dependent on the position of the same ray on objective $h_{\mathrm{ol}}$ through magnification $M_{\mathrm{TL}}$ between these two planes:

(.15) $$h_{\mathrm{TL}}=M_{\mathrm{TL}}h_{\mathrm{ol}}.$$

In case of an objective lens, the object is situated near the focus (coordinates $f_{\mathrm{ol}}$), thus it is possible to transform the previous equation to the form:

(.16) $$h_{\mathrm{TL}}=M_{\mathrm{TL}}f_{\mathrm{ol}}$$

and the contribution of a single transfer lens can be expressed in terms of the objective focal length [23]:

(.17) $$C_{c}=M_{\mathrm{TL}}^{2}\frac{f_{\mathrm{ol}}^{2}}{f_{\mathrm{TL}}}$$

According to this result, weak transfer lenses are recommended to reduce the chromatic aberration of the whole system. These theoretical conclusions were confirmed by computer simulations. Electrostatic and magnetic transfer lenses were investigated. The electrostatic lenses were used for a short focal length because of their possible miniaturisation, where the magnetic lenses were unfeasible due to obligatory construction parameters of the microscope.

**Fig. 7:**
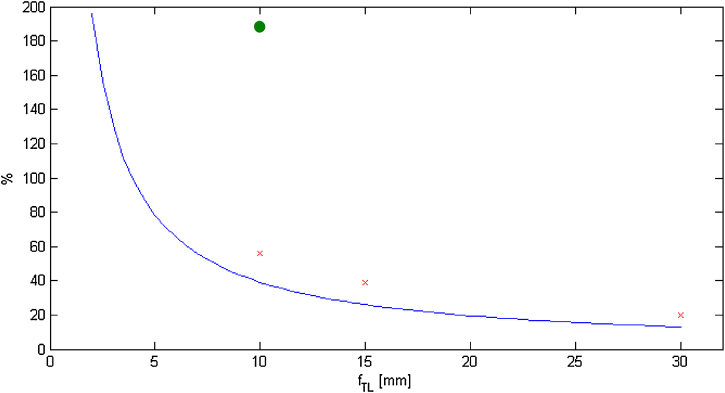
The comparison of the properties of the chromatic aberration corrector calculated by the computer simulation with the magnetic transfer lenses in two-doublets configuration (red crosses-permanent magnet transfer lenses, green dot-electrostatic case) and an analytical dependence (blue curve). (For interpretation of the references to color in text/this figure legend, the reader is referred to the web version of the article.)

The contribution to the total chromatic aberration depending on the focal length of a single transfer lens is shown in Fig. 7. The electrostatic lenses turned out to be inappropriate (Fig. 7), because they deteriorate the chromatic aberration much more than the magnetic ones. This critical deterioration by electrostatic doublets can be proved by comparison with results of $C_{c}$ calculation for a magnetic doublet with the same focal length. This behaviour is demonstrated on the example of a two-doublets configuration with a 10 mm focal length, which leads to an increase of the $C_{c}$ of 288% in the electrostatic case and only 166% in the magnetic case.

The consequences for the concept of a desktop corrected LVEM are very fundamental. The size of the corrector body with the appropriate weak transfer lenses would be comparable to the main body of the microscope column. It undoubtedly increases mechanical vibrations and thus increases requirements for vibration damping.

It should be emphasized that the chromatic aberration will still be a limiting factor despite the correction of the spherical aberration. A hexapole corrector is therefore suitable mostly for a low-voltage STEM application, in contrast to the TEM, where the energy spread is broadened by the passage of the beam through the sample.

The combination of a hexapole corrector with the proper monochromator could be the solution, with obvious advantages for spectroscopy. Because of the available degree of monochromatisation and the size of the instrument, it would be suitable only for LVEM25. The size of the column would be extended by approximately 100 mm by the monochromator, so the monochromator size is comparable with the LVEM5 column.

## Outline of the hexapole corrector design

**Fig. 8:**
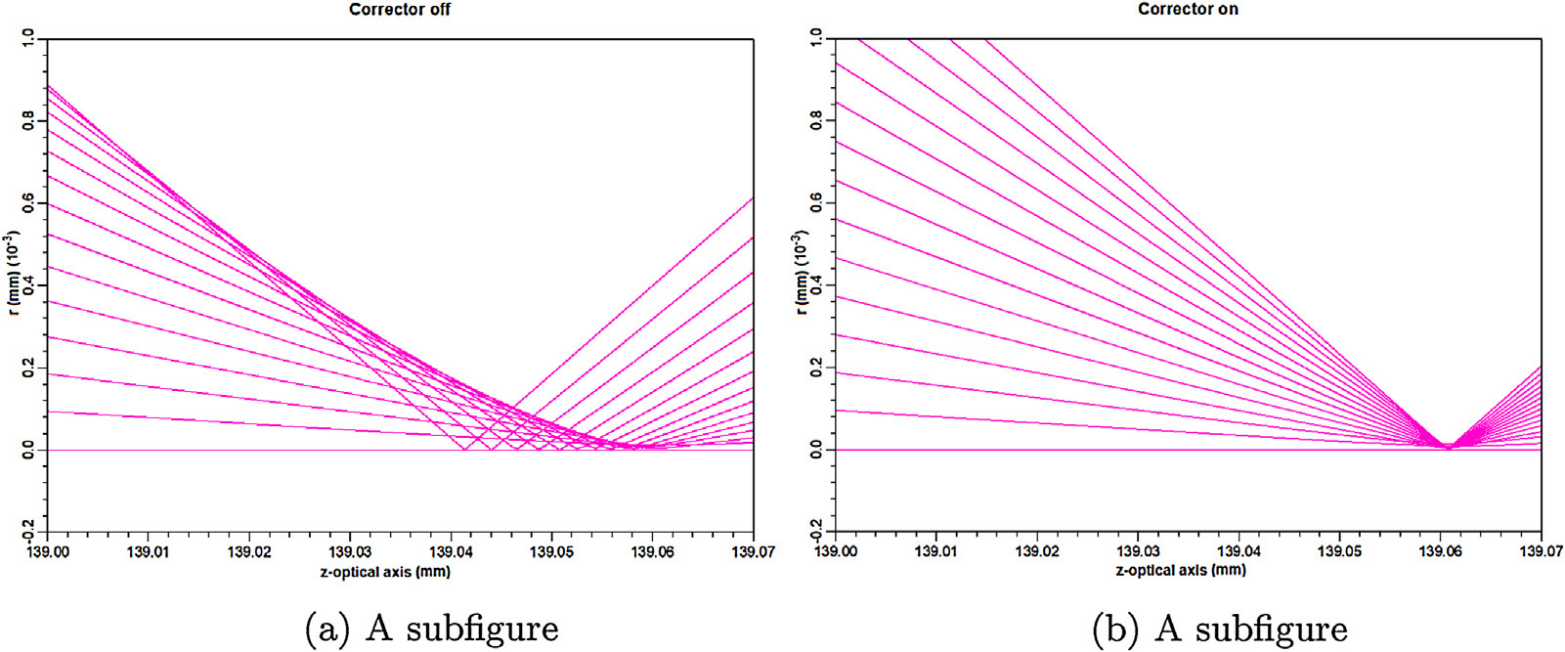
The details of the area near the intersection of the rays. The axis denoted *r* represents distance from the optical axis. Figure (a) shows the situation with hexapoles turned off and figure (b) demonstrates the corrected beam with an energy of 25 kV behind the simple magnetic lens. Correcting ability of the mentioned hexapole corrector is clearly visible by the size of the intersection.

The hexapole corrector has been designed with special attention to the maximum possible symmetry utilization. The considered design contains one doublet of the transfer lens, which is based on one set of permanent magnets with a magnetisation direction perpendicular to the optical axis. This configuration ensures the maximum possible symmetry. But the presence of correction coils would be unnecessary, due to the necessity for precise adjustment of transfer lenses and manufacturing limitations of the permanent magnets. It was proved by the calculations that the considered design of the corrector is not sensitive to gap size and bore diameter, which makes it easier to incorporate the corrector to the existing uncorrected LVEM25. The size of the corrector column is dependent on the focal length of the transfer lens. However, a focal length of a transfer lens approximately 30 mm seems to be an acceptable compromise between chromatic deterioration and column extension. It was proved that the presented hexapole corrector is capable of the spherical aberration correction (Fig. 8).

**Fig. 9:**
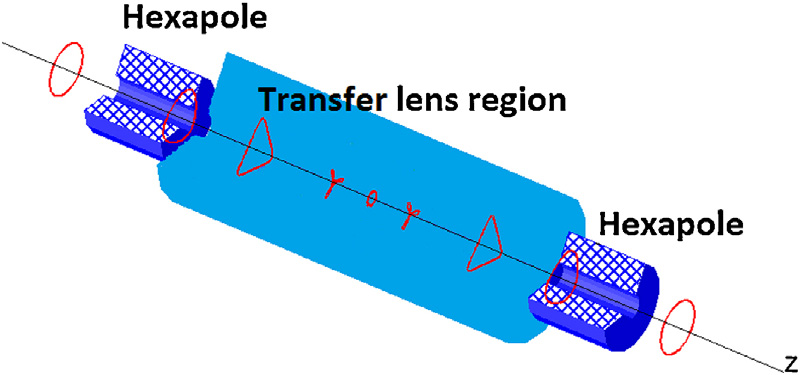
The beam caustic through the suggested hexapole corrector shown in schematic illustration.

The action of two hexapoles on the beam shape is shown in Fig. 9. The proper adjustment of the entire corrector guarantees a circular, slightly divergent beam with a “negative spherical aberration” at the end of the corrector.

The dimensions of an hexapole-doublet-hexapole system are approximately 140 mm in length and 30 mm in diameter for a 30 mm focal length of a single transfer lens.

## Conclusion

The aberration corrected LVEM is very promising technology, suitable for imaging of sensitive samples. In contrast to the conventional TEM, it provides more enhanced contrast, especially when observing samples containing light atoms, where achieving sufficient contrast is often challenging. The current development of these devices focuses on integration of the aberration corrector optimized for low-voltage systems. The initial analysis of feasibility and consideration of potential benefits has already been done. The presented study of aberration effects confirms the expected improvement of the spatial resolution, however there are still plenty of factors to be considered.

A hexapole corrector has been chosen due to its lower complexity, which is important for miniaturization for the desktop LVEM. Especially the influence of the chromatic aberration has appeared to be crucial. In addition, the unavoidable transfer lenses of a hexapole corrector increase the total chromatic aberration of the whole system. Magnetic lenses need to be used for this purpose due to their lower deterioration of the chromatic aberration, as opposed to using electrostatic ones, which deteriorate chromatic aberration severely. According to our calculations, the contribution of an electrostatic doublet to the chromatic aberration is almost 3 times higher. Due to this dramatic deterioration, we concentrated on magnetic doublets, more specifically those designed with permanent magnets because of the dimension parameters of the desktop LVEM.

The contribution of transfer lenses to the total chromatic aberration is more significant for lenses with a short focal length. A compromise between an acceptable size of the corrector and the influence of chromatic aberration was set at 15–30 mm as the focal length of a single transfer lens. According to all of these conditions, the main column of the corrector should be less than an acceptable 140 mm in length.

Magnetic lenses used for this purpose would require a special design based on the permanent magnets, because conventional magnetic lenses (even weak enough) that are suitable in terms of chromatic aberration, would be too massive and therefore they are not compatible with the corrector for the desktop LVEM.

An additional unwanted contribution of the unnecessary transfer doublet to the chromatic aberration can be only reduced by the above-mentioned proper system design. In principal it can not, however, be corrected by a hexapole corrector. To deal with the deterioration caused by the chromatic aberration, the energy spread has to be eliminated. The diameter of the aberration disc (neglecting chromatic aberration) is estimated at approximately 0.2 nm with a correction of the primary spherical aberration and 0.1 nm for the secondary spherical aberration. To keep the influence of the chromatic aberration below or at least comparable with the spherical aberration, it is necessary to reduce all possible unwanted contributions to the chromatic aberration. The reported maximum value of the energy spread is at the limit of attainability by current monochromatisation [20]. For these reasons it is more convenient to use the corrector for STEM application, because there is no additional broadening of the energy spread caused by an interaction with a sample. We expect that the described corrector incorporated to LVEM 25 will improve the resolution by 20%. The reported aberration-corrected LV-STEM will provide substantially enhanced contrast for light element samples making it convenient for biological sciences with no need for additional contrast-enhancing staining. The key application areas considered for this instrument are in virology, pathology and drug delivery research.

## References

[1] KRIVANEK O.L., LOVEJOY T.C., DELLBY N. (2015). Aberration-corrected STEM for atomic-resolution imaging and analysis. J. Microsc..

[2] Scherzer O. (1947). Sphärische und chromatische Korrektur von Elektronen-Linsen. Optik.

[3] Hawkes P.W. (2009). Aberration correction past and present.

[4] Delong Instruments LVEM 5. Available at: http://www.delong.cz/products/lvem5/ (accessed 29.06.17).

[5] Delong Instruments LVEM 25. Available at: http://www.delong.cz/products/lvem25/ (accessed 29.06.17).

[6] Linck M., Harttel P., Uhlemann S. (2016). Chromatic aberration correction for atomic resolution TEM imaging from 20 to 80 kV. Phys. Rev. Lett..

[7] Sasaki T., Sawada H., Hosokawa F. (2010). Performance of low-voltage STEM/TEM with delta corrector and cold field emission gun. J. Electron Microsc..

[8] CHang Wei-Yu, Chen Fu-Rong (2017). Development of compact Cs corrector for desktop electron microscope. Ultramicroscopy.

[9] Beck V.D. (1978). A hexapole spherical aberration corrector. Optik.

[10] Crewe A.V., Kopf D.A. (1980). A sextupole system for the correction of spherical aberration. Optik.

[11] Rose H., Wan W. (2003). Aberration correction in electron microscopy. IEEE Particle Accelerator Conference (PAC): Knoxville.

[12] Uhlemann S., Haider M. (1998). Residual wave aberrations in the first spherical aberration corrected transmission electron microscope. Ultramicroscopy.

[13] Rose H. (2004). Outline of an ultracorrector compensating for all primary chromatic and geometrical aberrations of charged-particle lenses. Nucl. Instrum. Methods Phys. Res. Sect. A: Accel. Spectrom. Detect. Assoc. Equip..

[14] ROSE H. (2005). Prospects for aberration-free electron microscopy. Ultramicroscopy.

[15] Orloff J. (2009). Handbook of Charged Particle Optics.

[16] van den Bos A. (2000). Aberration and Strehl ratio. J. Opt. Soc. Am. A.

[17] Mahajan V.N. (1982). Strehl ratio for primary aberrations: some analytical results for circular and annular pupils. J. Opt. Soc. Am..

[18] Intaraprasonk V., Huolin L., XIN, MULLER D.A. (2008). Analytic derivation of optimal imaging conditions for incoherent imaging in aberration-corrected electron microscopes. Ultramicroscopy.

[19] Chang L.Y., Kirkland A.I., Titchmarsh J.M. (2006). On the importance of fifth-order spherical aberration for a fully corrected electron microscope. Ultramicroscopy.

[20] Mankos M., Shadman K., Kolarik V. (2016). Novel electron monochromator for high resolution imaging and spectroscopy. J. Vac. Sci. Technol. B Nanotechnol. Microelectron.: Mater. Process. Meas. Phenom..

[21] Hawkes P.W. (1982). Magnetic electron lenses.

[22] Hawkes P.W., Kasper E. (1996). Principles of Electron Optics.

[23] Haider M., Müller H., Uhlemann S. (2008). Advances in Imaging and Electron, Volume 153, Hawkes ed..

